# Identification of α(1,6)fucosylated proteins differentially expressed in human colorectal cancer

**DOI:** 10.1186/1471-2407-11-508

**Published:** 2011-12-07

**Authors:** Laura Muinelo-Romay, Susana Villar-Portela, Elisa Cuevas, Emilio Gil-Martín, Almudena Fernández-Briera

**Affiliations:** 1 Department of Biochemistry, Genetics and Immunology, Faculty of Biology, University of Vigo, Campus As Lagoas-Marcosende S/N, 36310, Vigo, Spain; 2 Pathology Service, University Complex Hospital of Ourense, Ramón Puga 54, 32005, Ourense, Spain

**Keywords:** Colorectal cancer, Glycoproteins, α(1,6)fucosyltransferase, GRP94, IgGFcBP

## Abstract

**Summary:**

**Background**

A universal hallmark of cancer cells is the change in their glycosylation phenotype. One of the most frequent alterations in the normal glycosylation pattern observed during carcinogenesis is the enhancement of α(1,6)linked fucose residues of glycoproteins, due to the up-regulation of the α(1,6)fucosyltransferase activity. Our previous results demonstrated the specific alteration of this enzyme activity and expression in colorectal cancer, suggesting its implication in tumour development and progression.

**Methods**

In the current work we combined a LCA-affinity chromatography with SDS-PAGE and mass spectrometry in order to identify α(1,6)fucosylated proteins differentially expressed in colorectal cancer. This strategy allowed the identification of a group of α(1,6)fucosylated proteins candidates to be involved in CRC malignancy.

**Results**

The majority of the identified proteins take part in cell signaling and interaction processes as well as in modulation of the immunological response. Likewise, we confirmed the increased expression of GRP94 in colorectal cancer tissue and the significant down-regulation of the IgGFcBP expression in tumour cells.

**Conclusion**

All these results validate the importance of *core-*fucosylated proteins profile analysis to understand the mechanisms which promote cancer onset and progression and to discover new tumour markers or therapeutic targets.

## Background

Colorectal carcinoma (CRC) is one of the most frequent tumours in the Western world. Although early stages are successfully treatable, many cases are undiagnosed until late stages when the prognosis is poor [1]. Therefore, the identification and verification of proteins that have a functional role in the patho-physiology of CRC remains an important goal to discover new biomarkers for diagnosis, prognosis and follow-up, as well as to find therapeutic targets.

Glycosylation plays fundamental roles in controlling various biological processes such as embryonic development, immune response and cell-cell interactions involving sugar-sugar or sugar-protein specific recognition [2]. An universal hallmark of cancer cells is the change in their glycosylation phenotype, with several effects on this tumour cells behavior [3]. In this sense, glycosylation analysis has become an important target for proteomic research and has reached great interest to understand the molecular events associated with tumour development and progression.

One of the most frequent alterations in the normal glycosylation pattern observed during carcinogenesis is the enhancement of α(1,6)linked fucose residues of glycoproteins [4,5]. The enzyme responsible of this fucosylation step is the α(1,6)fucosyltransferase [FUT8, α(1,6)FT], which catalyzes the transfer of a fucose residue from GDP-fucose to the innermost GlcNAc of hybrid and complex N-linked oligosaccharides via α(1,6)linkage. Numerous studies have demonstrated the key role of α(1,6)fucosylation in the activity of proteins strongly implicated in both tumour growth, such as EGFR, TGFR-β_1 _or VEGFR-2 [6-8], and tumour dissemination, for example, E-cadherin or α3β1 and α5β1 integrins [9-11]. In addition, several α(1,6)fucosylated proteins have been proposed as potential biomarkers for different types of tumours. The most representative case is the α(1,6)fucosylated fraction of α-fetoprotein (AFP-L3), currently employed as a specific tumour marker for hepatocarcinoma (HCC) [12]. Other aberrantly *core*-fucosylated proteins such as GP73, haptoglobin (Hp), transferrin, α-1-acid glycoprotein or α-1-antitrypsin, have also been reported as promising serum biomarkers for HCC [13,14]. Moreover, an important elevation of serum fucosylated forms of Hp and RNase1 has been described in pancreatic tumours [15,16].

The identification and validation of specific tissue and/or serum α(1,6)fucosylated proteins which share altered expression levels is of great value to clarify the insights of the critical events in cancer progression. However, the proteomic analysis of glycoproteins is tedious because it requires their isolation from complex biological samples that contain both non glycosylated and very heterogeneously glycosylated proteins [17]. Thus, the application of different analytic approaches has revealed as the most efficient tool to study the tissue or serum profile of these proteins. In this sense, previous studies have achieved the affinity purification of α(1,6)fucosylated proteins using lectins that specifically recognize the (1,6)fucose linkage, such as LCA (*Lens culinaris agglutinin*) [18] or AAL (*Aleuria aurantia lectin*) [19], followed by separation methods (HPLC or SDS-PAGE) and analytical LC-MS/MS to identify the proteins differentially expressed and/or fucosylated in cancer tissue [20,21].

We have previously reported the specific alteration of α(1,6)FT activity and expression in CRC, suggesting its implication in tumour development and progression [22,23]. To complete the knowledge about the importance of the α(1,6)fucosylation in CRC, in the present study we have undertaken an affinity enrichment approach to compare the α(1,6)fucosylated proteins profile of healthy and tumour colorectal tissues. This strategy has allowed us the identification of several α(1,6)fucosylated proteins as candidates to be involved in CRC malignancy. In particular, we observed a significant alteration of GRP94 and IgGFcBP expression levels in colorectal cancer tissue. These results validate the importance of *core-*fucosylated proteins profile analysis in CRC as a way to discover new tumour markers or therapeutic targets.

## Methods

### Human tissue samples

Paired colorectal tissue samples, both from tumour and adjacent healthy mucosa, were obtained from patients with CRC who underwent surgery at the University Complex Hospital of Ourense, Spain. Mucosa was considered healthy if distant by at least 10 cm from the tumour. The approval of the appropriate local Institutional Review Board as well as the informed consent from the patients, were obtained.

The specimens used for immunohistochemical analysis were fixed in formalin (10%), embedded in paraffin (60°C) and subjected to hematoxylin and eosin staining (standard procedure) for evaluating their microscopic features. The specimens employed for the chromatography enrichment and Western/Lectin blot analysis were washed with ice-cold saline buffer and stored frozen at -85°C until use.

### Protein extraction

Colorectal tissue was homogenized in 6 vol. of 0.01 M Tris-HCl buffer (pH 7.4), containing 0.25 M sucrose, 1% Triton X-100 and a cocktail of protease inhibitors (Complete™ Mini tablets, Roche). The homogenate was centrifuged for 10 min at 15000*xg *and 4°C. The supernatant obtained was recovered and the protein concentration of this final preparation (total fraction, TF) was determined by BCA protein assay, using bovine serum albumin as standard. The tissue extract was stored at -20°C until use.

### α(1,6)fucosylated proteins enrichment with LCA-affinity chromatography

LCA-affinity chromatography was performed as previously described by Dai *et al. *[21] with some modifications. LCA column was prepared by adding 4 mL of agarose-bound lectin (Sigma-Aldrich Aldrich) into polypropylene columns (Econo Column, Bio-Rad). The columns were equilibrated *ab initio *with Tris-HCl 0.05 M (pH 7.2). Once equilibrated, 1 mL of the total protein (TF) preparation was loaded onto the LCA column. After incubating for 10 min, the unbounded proteins (FI) were washed out with 15 mL of equilibrating buffer. The unspecific retained proteins (FII) were then removed with 10 mL of Tris-HCl 0.05 M (pH 7.2) containing NaCl 0.3 M. Finally, the specific bounded fraction (FIII) was eluted using 10 mL of Tris-HCl 0.05 M (pH 7.2) containing NaCl 0.3 M, α-methyl-D-mannopyranoside 0.4 M and α-methyl-D-glycopyranoside 0.4 M. The chromatographic process was controlled by spectrophotometry at 280 nm with a UVIKON 930 spectrophotometer (Kontron Instruments). All fractions obtained were then centrifugated in Amicon Ultra-4 Centrifugal Filters Devices (Millipore) for concentrating and desalting. The protein recovery of the lectin column was determined by means of the BCA protein assay, using bovine serum albumin as standard.

### One-dimensional electrophoresis and lectin blot

20 and 10 μg of protein from total extraction (TF) and FI, FII and FIII chromatography fractions were subjected in parallel to SDS-PAGE (12% polyacrylamide gel) under reducing conditions. The gel loaded with 20 μg of protein was stained with Coomassie Brilliant Blue R-250 (Bio-Rad). The proteins of the gel loaded with 10 g of protein were electrotransferred onto a polyvinylidene difluoride membrane (Hybond-P, Amersham Bioscience). After blotting, the membrane was washed with TBS containing 0.05% Tween-20 (T-TBS) (v/v), blocked with 3% (w/v) dried serum albumin in T-TBS overnight at 4°C, and incubated with 1/2,000 biotinylated-LCA (Vector Laboratories) for 1 h at room temperature. After washing with T-TBS, the blot was incubated with 1/2,000 diluted Streptavidin-Alkalin phosphatase (Sigma-Aldrich) for 1 h at room temperature. Finally, the blot was washed with T-TBS and the colour was developed using BCIP/NBT Liquid Substrate System (Sigma-Aldrich).

The band pattern and the intensity were obtained by densitometric analysis (GS-800 Calibrated Densitometer, Bio-Rad) using the Quantity One software for PC (Bio-Rad). The relative intensity of FIII bands was employed for comparative analysis between healthy and tumour mucosa.

### Protein identification by mass spectrometry

Differentially expressed bands present in FIII were excised from gels and sent for analysis to the Proteomics and Mass Spectrometry Facility at Parc Científic de Barcelona (Barcelona, Spain).

Proteins were in-gel digested with trypsin (Sequencing grade modified, Promega) in the automatic Investigator ProGest robot of Genomic Solutions. Briefly, excised gels spots were washed sequentially with ammonium bicarbonate buffer and acetonitrile. Proteins were reduced and alkylated by treatment with 10 mM DTT solution during 30 min, and treatment with a 55 mM solution of iodine acetamide, respectively. After sequential washings with buffer and acetronitrile, proteins were digested overnight at 37°C with 0.27 nmol trypsin. Tryptic peptides were extracted from the gel matrix with 10% formic acid and acetonitrile; the extracts were pooled and dried in a vacuum centrifuge.

Tryptic peptides were analyzed by nano-electrospray ionization MS/MS (ESI-Q-TOF) with a Q-TOF-Global (Micromass-Waters). Samples were resuspended in 12 μL of a 10% formic acid solution, and 4 μL were injected for chromatographic separation onto a reverse-phase capillary C_18 _column (75 μm of internal diameter and 15 cm of length, PepMap column, LC Packings). The eluted peptides were ionized via coated nano-ES needles (PicoTip™, New Objective). A capillary voltage of 1,800-2,200 V was applied together with a cone voltage of 80 V. The collision in the CID (collision-induced dissociation) was 20-35 eV with argon as collision gas. Data were generated in a PKL file format, which were submitted for database searching in the MASCOT server.

### Western blot validation

20 μg of total protein preparation were subjected to SDS-PAGE (12% polyacrylamide gel) and electrotransferred onto a polyvinylidene difluoride membrane (Hybond-P, Amersham Bioscience). After blotting, the membrane was washed with TBS containing 0.05% Tween-20 (T-TBS) (v/v). The SNAPi.d. protein detection System (Millipore) was employed for the immunodetection. The membrane was blocked with 1% (w/v) serum albumin in T-PBS for 10 min and incubated with primary antibodies [mouse anti-GRP94 monoclonal antibody (3/1,000, Abcam), mouse anti-pIgR monoclonal antibody (3/1,000, Santa Cruz Biotechnology) and rabbit polyclonal anti-IgGFcBP (1/500, Sigma-Aldrich)] for 10 min at room temperature. After a wash with T-TBS, the blot was incubated with 1/500 diluted secondary antibody, an alkalin-phosphatase-conjugated antibody against mouse or rabbit IgG (Dako Cytomation), for 10 min at room temperature. Finally, the blot was washed with T-TBS and the colour was developed using BCIP/NBT Liquid Substrate System (Sigma-Aldrich).

### Immunohistochemistry

Tissue sections (2-3 μm) were deparaffinized in xylene, rehydrated in a graded ethanol series and incubated in citrate buffer for 10 min in a microwave. Endogenous peroxidase activity was blocked with 0.5% (v/v) hydrogen peroxide in methanol. After a rinse in PBS, bovine serum was applied to block non-specific interactions. Sections were then incubated 1 h with the primary antibody (rabbit polyclonal anti-IgGFcBP, 1/50, Sigma-Aldrich) at room temperature. After a rinse in PBS, sections were incubated 10 min with the primary antibody enhancer, and 1 h at room temperature with the secondary antibody bound to peroxidase. The peroxidase reaction was visualized by incubating with DAB (3,3'-diaminobenzidine). Finally, after a wash in water, the sections were counterstained with haematoxylin, dehydrated in a graded ethanol series, washed in xylene and mounted on a glass slide. Negative controls were performed using PBS instead of the primary antibody. The semiquantitative staining analysis was performed by expert pathologists.

### Statistical analyses

Statistical analyses were performed using SPSS v. 15.00 for WINDOWS XP. The results were considered significant when *p*≤0.05.

## Results

### Enrichment of α(1,6)fucosylated proteins from colorectal tissues by LCA-affinity chromatography

**Figure 1:**
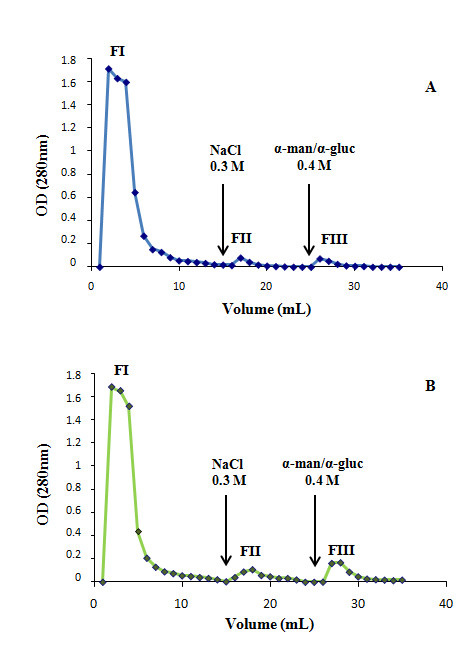
**Chromatographic profile. **Representative chromatographic profile of α(1,6)fucosylated proteins enrichment from healthy (A) and tumour (B) colorectal specimens.

The success of a comparative glycoproteomic approach resides in the quality of the enrichment process. Therefore, lectin affinity chromatography protocols are commonly used to isolate different types of glycoproteins. In the present study, the lectin LCA, which recognizes α-mannopyranosyl and α-glucopyranosyl terminal residues of α(1,6)fucosylated proteins [18], was employed to separate this group of proteins. After chromatography, we obtained three distinct fractions: FI (which contains non-retained proteins), FII (composed of unspecific retained proteins and FIII, mainly enriched in α(1,6)fucosylated proteins). Five paired samples of healthy mucosa and tumour tissue were submitted to this prefractionation procedure, being the chromatography profile (Figure 1A and 1B) and the protein recovery very similar from both tissues (Table 1).

**Table 1:** Protein recovery during LCA-affinity chromatography

		TF	FI	FII	FIII
**Healthy**	**Mean (mg) ± SEM**	4.83 ± 0.67	2.54 ± 0.22	0.21 ± 0.07	0.33 ± 0.04
***n *= 5**	**Percentage (%)**	100	52.62	4.27	6.81

**Tumour**	**Mean (mg) ± SEM**	5.38 ± 0.33	3.03 ± 0.50	0.28 ± 0.06	0.44 ± 0.05
***n *= 5**	**Percentage (%)**	100	56.24	5.19	8.23

SEM, standard error of the mean

**Figure 2:**
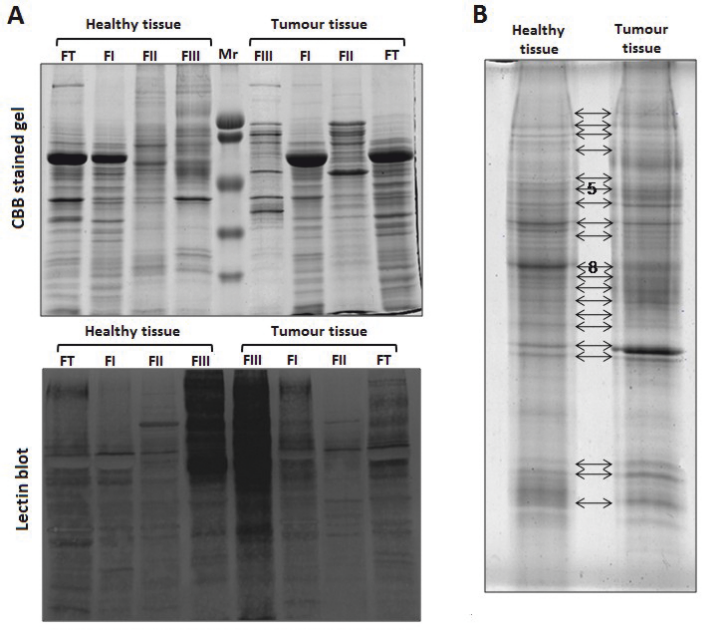
**Different expression of α(1,6)fucosylated proteins in healthy and tumour colorectal tissues. **(A) Representative Coomassie brilliant blue stained 1D-SDS-PAGE gel of the total protein fraction (TF) and the following chromatographic fractions obtained; FI (non-retained proteins), FII (unspecific retained proteins) and FIII [α(1,6)fucosylated proteins] and the corresponding lectin blot in a pair of healthy and tumour tissue. (B) Patern of bands obtained in the FIII fraction of a pair of healthy and tumour samples. Bands 5 and 8 were excised and submitted to mass spectrometry (MS) analysis.

Proteins from the crude extract (TF) and the chromatography fractions were analyzed by means of 1D-SDS-PAGE and lectin blot. The majority of the proteins observed in TF were eluted in FI (Figure 2, left). In both fractions we detected a strong band of about 60 kDa corresponding to albumin. The elimination of the albumin-associated band (a non-glycosylated protein), allowed the visualization of minority proteins in FII and FIII (Figure 2A). Additionally, in the fraction enriched with α(1,6)fucosylated proteins, FIII, bands not visualized in the previous fractions were observed (Figure 2B), as well as others hardly expressed in FI and FII (Figure 2C). This electrophoretic profile ratified the appropriate functioning of the prefractionation procedure. Likewise, the lectin blot confirmed the enrichment of α(1,6)fucosylated proteins in FIII, since a higher number of LCA-reactive bands was observed in comparison to the other fractions (Figure 2, right).

### Comparison of 1D-SDS-PAGE pattern of α(1,6)fucosylated proteins from healthy and tumour colorectal mucosa

With the aim to identify the α(1,6)fucosylated proteins differentially expressed in healthy and tumour colorectal mucosa, the band profile of the FIII fraction after the 1D-SDS-PAGE was analyzed using the Quantity One Software (Bio-Rad). The analysis compared the intensity level of the detected and matched bands from paired tumour/healthy specimens from 5 CRC patients. A total of 20 bands were compared between healthy and tumour mucosa. Although no statistically significant differences were found, some bands showed a clear trend of increase or decrease in tumour samples. In this sense, our interest was focused in two bands, n° 5 and n° 8 (Figure 2A). The first one showed a higher intensity in tumour compared to healthy mucosa (+4.57 fold-change) in 4 of 5 patients, while the second one showed a lower tumour intensity in 3 of 4 patients (-18.80 fold-change). The two bands were excised and submitted to mass spectrometry (MS) analysis.

### Identification of α(1,6)fucosylated proteins by mass spectrometry

The CapLC-n-ESI-Q-TOF method allowed us the identification of proteins present in the n° 5 and n° 8 bands. The identity of these proteins and other characteristics, such as the experimental relative molecular mass (Mr), the score and the SwissProt/NCBInr accession number, are summarized in Table 2. For the band n° 5, we found 5 candidate proteins with a score value higher than 37 (the limit to consider the identification of the protein not accidental). The protein with the highest score was the glucose regulated glycoprotein of 94 kDa (GRP94), an endoplasmic reticulum chaperone which participates in the maintenance of the cell homeostasis, specially under stress conditions. The second candidate was the polymeric Ig receptor (pIgR), responsible for the transport of polymeric immunoglobulins (IgA and IgG) across the mucose epithelial cells. The others candidates were the adhesion vascular protein 1 (VAP-1), the fibulin-1C and the platelet glycoprotein IIIa (GP IIIa), implicated in cell adhesion, recognition process and the immune response, respectively.

**Table 2:** Identification of α(1,6)fucosylated proteins by mass spectrometry

Protein	Band n°	Mr (kDa)	NCBInr Swiss-Prot accession n°	Function	Score	Coverage rate (%)
**Glucose regulated glycoprotein of 94 kDa**	5	92.7	B4DHT9	Stress response/chaperone	743	20

**Polymeric immunoglobulins receptor**	5	84.4	PO1833	Immune response	383	10

**Vascular adhesión protein 1**	5	85.1	Q16853	Cell adhesion molecule/Immune response	361	11

**Fibulin-1 C**	5	78.5	P23142-4	Extracellular matrix component/cell adhesion and migration	339	12

**Platelet glycoprotein IIIa**	5	87.4	PO5106	Cell adhesion molecule/Immune response	156	5

**IgG Fc binding protein**	8	59.6	Q9Y6R7	Cell adhesion molecule/Immune response	1320	6

**Unknown protein product**	8	53.8	Q6ZVX0	unknown	343	24

**Immunoglobulin G B12, heavy chain**	8	51.2	gi|15825648	Cell adhesion molecule/Immune response	272	16

**Immunoglobulin α-1, constant region**	8	54.3	P01876	Cell adhesion molecule/Immune response	248	14

Each protein score represents the sum of the score of each ion protein analyzed and this is, in turn considered numerically as -10 * log (*p*), where *p *is the probability that the matching was made randomly and coverage rate (%) represents the percentage of the total protein sequence that overlap with the peptide fragments.

On the other hand, the highest scored candidate protein integrating the band n° 8 was the IgG Fc binding protein (IgGFcBP), a secreted protein present in the colon mucus. The other two proteins identified as potential candidates were the heavy chain of IgGB12 and the Fc region of IgA-1, both implicated in the modulation of the cell immune mechanisms.

All of these candidate proteins are glycosylated and some of them, such as pIgR, IgGFcBP, VAP-1 and the GP-IIIA, have been previously described as α(1,6)fucosylated proteins, therefore validate the efficiency of the chromatography approach performed for the fractionation of specifically glycosylated proteins [18].

### Validation of GRP94, pIgR and IgGFcBP alteration by Western blot

**Figure 3:**
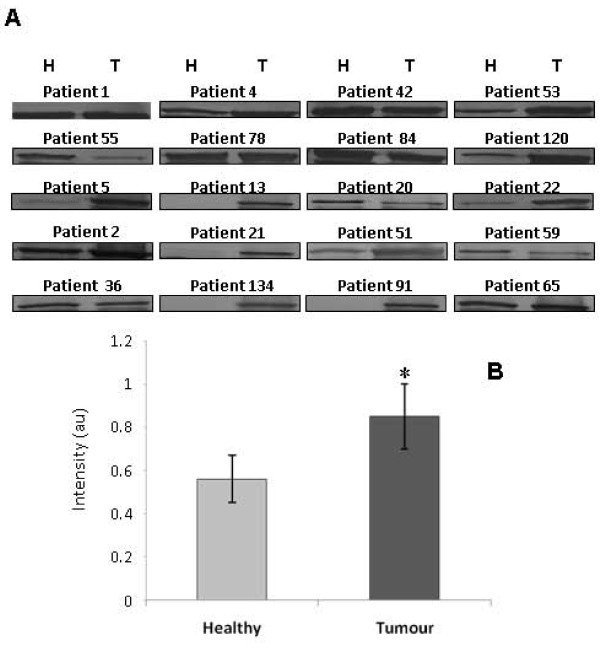
**GRP94 expression in healthy and tumour colorectal tissues. **(A) Validation of the 1D-SDS-PAGE results for GRP94 by immunoblotting in 20 paired samples of healthy (H) and tumour (T) tissue. (B) Statistically significant increase of the GRP94 expression found in tumour tissue. *p < 0.05, according to Wilcoxon's test.

**Figure 4:**
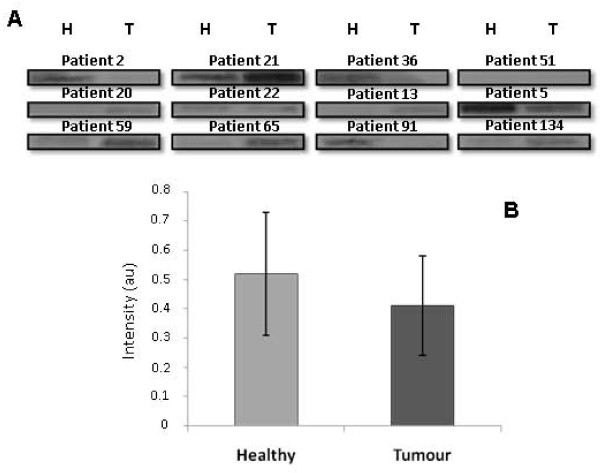
**pIgR expression in healthy and tumour colorectal tissues. **(A) Validation of the 1D-SDS-PAGE results for pIgR by immunoblotting in 12 paired samples of healthy (H) and tumour (T) tissue. (B) The statistical analysis showed no significant differences between healthy and tumour tissues according to Wilcoxon's test.

**Figure 5:**
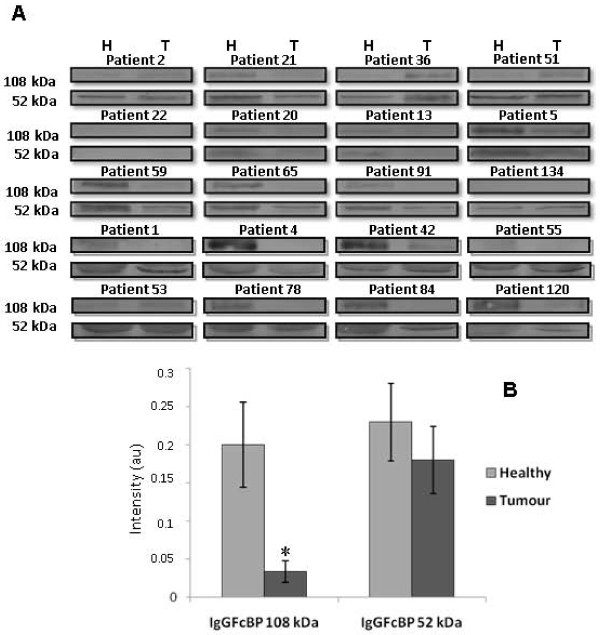
**IgGFcBP expression in healthy and tumour colorectal tissues. **(A) Validation of the 1D-SDS-PAGE results for IgGFcBP by immunoblotting in 20-paired samples of healthy (H) and tumour (T) tissue. The Western blot allowed the visualization of two underexpressed isoforms of ~108 and ~52 kDa. (B) For the ~108 kDa band, this decrease was statistically significant. *p < 0.05, according to Wilcoxon's test.

In order to validate the results obtained by 1D-SDS-PAGE, three proteins, GRP94, pIgR and IgGFcBP were chosen to determine their expression in tumour and healthy neighboring tissue. This selection was performed taking into account their score and their possible implication in CRC development and/or progression. The total protein fraction (TF) of 20 paired samples was employed for Western blotting detection of GRP94 and IgGFcBP, while for pIgR we examined 12 patients. The analysis demonstrated higher levels of GRP94 expression in the tumour tissue in 15 patients of the total analyzed (*p *= 0.01, according to Wilcoxon's test, Figure 3). On the other hand, the pIgR was identified in the same band as GRP94, which showed a higher intensity in tumour tissue. However, after the specific immunodetection of pIgR, no differences were observed between the tumour and healthy mucosa (*p *= 0.87, according to Wilcoxon's test, Figure 4). Finally, the Western blot for IgGFcBP allowed the visualization of two bands, corresponding to 108 and 52 kDa (Figure 5). The expression of both isoforms decreased in the tumour tissue of 13 and 12 patients, respectively, being this decrease statistically significant for the 108 kDa isoform (*p *= 0.005 and *p *= 0.191 according to Wilcoxon's test) (Figure 5). It is also important to highlight that IgGFcBP was identified in an electrophoretic band with lower levels of intensity in tumour tissue.

### Immuhistochemical analysis of IgGFcBP

**Figure 6:**
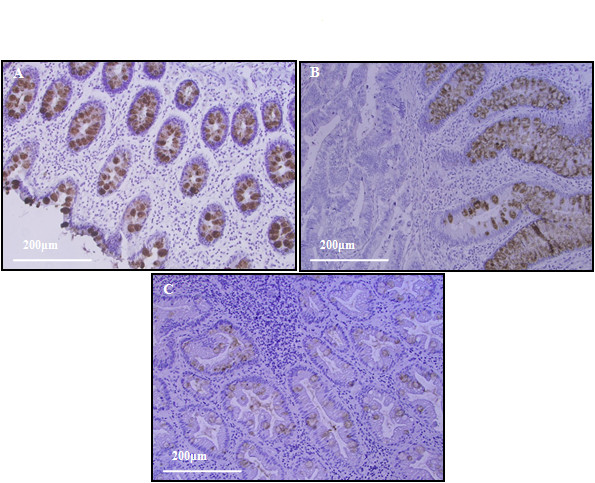
**Immunohistochemistry analysis of IgGFcBP expression**. (A) Healthy colon tissue with a specific IgGFcBP staining localized in the intracellular mucus of globelet cells. (B) CRC tissue with a positive IgGFcBP staining present in the non-invasive zone. (C) Mixed hyperplastic/adenomatous polyp with the IgGFcBP positive glands in the non dysplastic areas.

Since the IgGFcBP histology distribution in CRC has not been studied previously in order to assess how specific is the alteration of its expression during the carcinogenesis process 20 paired specimens of healthy and tumour mucosa as well as 6 polyps, were analyzed by immunohistochemistry. The presence of the protein was detected as a brown staining clearly localized in the intracellular mucus of goblet cells (Figure 6A). A positive IgGFcBP expression was observed in all the healthy specimens (Figure 6A), while in tumour only 3 cases (early stage CRC) were positive (Figure 6B). In these positive tumours, the IgGFcBP expression was confined to the non-infiltrating epithelia. In addition, although all tumour analyzed are no mucilaginous a higher mucinous component was observed in the 3 cases with positive IgGFcBP expression. The different IgGFcBP immunohistochemical expression in tumour *vs*. healthy tissue was statistically significant (*p *= 0.001, according to Wilcoxon's test). Furthermore, 4 of the polyps analyzed expressed the IgGFcBP; one hyperplastic, two adenomatous, and one mixed of hyperplastic/adenomatous. Interestingly, the positive glands were placed in the non-dysplastic zones (Figure 6C).

## Discussion

The scientific community shows an increasing interest in qualitative and quantitative alterations that different glycoproteins undergo during the malignant transformation process. This interest is fundamentally focused on the detection of new tumour biomarkers for diagnosis, prognosis and follow-up of the disease, as well as on the development of new therapeutic targets. Thus, a large number of biomolecules currently used as tumour markers are glycoproteins [24,25].

In particular, the study of the α(1,6)fucosylated proteins expressed in tumour cells has achieved high importance. Several researchers have reported the specific alteration of α(1,6)FT activity and expression in malignant processes, such as HCC [26,27], thyroid papillary carcinoma [28] and ovarian adenocarcinoma [29]. Recently, we have also reported the alteration of the enzyme in CRC [22] and its implication in the disease progression (submitted data). In this sense, it is important to mention the critical role of α(1,6)FT activity in the modulation of different growth factor receptors such as EGFR, TGFβR or VEGFR. These molecules, strongly implicated in the carcinogenesis process and considered gold therapeutic targets for the treatment of solid tumours, need to be *core*-fucosylated to activate their intracellular signaling pathways [6-8]. Likewise, recent reports have shown that α(1,6)fucosylation of different adhesion molecules, such as adhesins or integrins, modify their functional activity, and could probably act as relevant factors for the acquisition of the migratory phenotype by the epithelial tumour cells [6,10,11]. Therefore, the identification and characterization of *core*-fucosylated proteins differentially expressed in tumour cells is considered of great usefulness to discover new tumour biomarkers and therapeutic agents.

In the present study we combined a LCA-affinity chromatography with SDS-PAGE and mass spectrometry in order to identify α(1,6)fucosylated proteins differentially expressed in the tumour tissue of 5 CRC patients. We identified a group of proteins candidates to be α(1,6)fucosylated and specifically regulated in colorectal tumours. Validating our approach, all the identified proteins have been described as glycoproteins, and most of them as α(1,6)fucosylated proteins. We selected three of them, the GRP94, the pIgR and the IgGFcBP in order to validate their altered expression during colorectal carcinogesis.

The GRP94 or endoplasmin is the most abundant glycoprotein in the endoplasmic reticulum. It belongs to the family of the heat-shock proteins, and together with GRP78 assists the folding and assembly of a wide range of proteins. The GRP94 also shows ATPase activity and plays an essential role in the cellular protection against different stress situations. Under pathological conditions, such as tumour growth, GRP94 is dramatically up-regulated as a survival mechanism [30], being its overexpression associated with a more aggressive tumour phenotype and a poor evolution of the disease [31,32]. In CRC, gene and protein GRP94 expression are strongly increased in both animal models and human tumours [33,34], and therefore GRP94 has been proposed as a useful diagnostic and prognostic marker for the disease. Concordantly, after an immunoblot analysis of the GRP94 expression in paired healthy and tumour colorectal specimens, we observed a significant increase in tumour tissue. In this sense, it is conceivable that up-regulation of GRP94 allows the correct folding of several oncogenic products promoting the colorectal carcinogenesis, although this increase has also been related with the acquisition of survival mechanisms by tumour cells to stand up lethal conditions, such as glucose starvation and hypoxia, and with the ability to form new distant solid tumours [30]. It also important to remark that although this protein is known to be glycosylated our study suggest for the first time its α(1,6)fucosylated status, however further studies should be developed to demonstrate this it.

In the same band where GRP94 was detected, MS analysis also identified the potential presence of pIgR. This glycosylated receptor is located in the basolateral membrane of the oral and gastrointestinal epithelial cells. Its main function is the transport of IgA and IgM polymeric forms across the epithelial cell membranes [35]. Recently, the α(1,6)fucosylation of this protein has been demonstrated in both, healthy and tumour hepatic specimens [36], confirming the results obtained in our study for colonic tissue. Interestingly, the importance of pIgR is not restricted to immunology, since changes in its expression, either increase or decrease, have been described in different types of tumours [37]. However, after our comparative immunoblot analysis between paired specimens of healthy and tumour mucosa, no significant differences were found in spite of the previous studies indicating an early decrease of pIgR expression during colorectal carcinogenesis [38]. It seems that the absence of pIgR in the tumour cells could induce a depletion of the immune response that promotes their malignant potential; nevertheless, its role in cancer development remains unknown.

Finally, we analyzed by Western blot and immunohistochemistry the expression of IgGFcBP, a protein secreted by the mucosa epithelial cells and present in the body fluids [31]. It specifically recognizes the constant fraction of IgG, and plays a relevant role in the structural maintenance of the mucosa through the binding to the MUC2 mucin [39]. Despite the theoretical molecular mass of IgGFcBP is ~500 kDa [31], SDS-PAGE reports Mr ~100-80 kDa depending on the tissue analyzed. In our study two bands of 108 and 52 kDa were detected, suggesting a tissue specific proteolytic process. Interestingly, the two bands showed a clear decrease in tumour specimens, statistically significant for the 108 KDa band. These results were confirmed by immunohistochemistry, since a positive IgGFcBP expression was observed in all the healthy specimens analyzed, while only 3 early staged tumours were found positive (with the expression mainly confined to the non-infiltrating epithelia). Concordantly with the specific expression of IgGFcBP in the goblet cells of the mucosa, the 3 positive tumours present higher percentage of mucinous component than the other specimens. It should be of great interest check the status of the IgGFcBP expression in mucilaginous tumours to demonstrate the independence between the tumour histology and the IgGFcBP downregulation. Furthermore, the analysis of 6 polyps showed expression of IgGFcBP in 4 of the specimens. Supporting our results, the lost of expression of this α(1,6)fucosylated protein in tumour specimens has also been reported in hepatic tissues [36] and the same tumour decrease of IgGFcBP has been described in animal models of CRC and human polyps [40,41]. The biological significance of the down-regulation of IgGFcBP in tumour cells is uncertain. In this sense, the binding between this protein and IgG could protect it from the action of bacterial proteases [31], promoting the immunological response of the mucosa. Thus, the IgGFcBP overexpression has been detected in ulcerous colitis and Cröhn patients [42]. Consequently, it is thought that low secreted levels of this protein in tumour cells could facilitate the immune evasion. However, the absence of IgGFcBP in the colon mucosa could also lead to the structural disorganization of intestinal mucus, promoting the exposure of mucosa to carcinogenic agents and, therefore, the appearance of premalignant lesions in the epithelium. Taking into account that increased levels of serum IgGFcBP have been observed in inflammatory processes, a possible decrease in CRC could be of great value as a biomarker for this neoplasia.

## Conclusion

In conclusion, the present study confirmed the altered expression of GRP94 in CRC and demonstrated for the first time its *core*-fucosylated status. Besides, a strong down-regulation of the IgGFcBP associated to the dysplastic phenotype acquisition was determined in pre-cancerous and cancerous lesions of colorectal mucosa. Despite the role of both molecules in CRC must be studied in depth, these results ratify the utility of screening α(1,6)fucosylated proteins differentially expressed in tumour colorectal mucosa as a way to identify molecules implicated in CRC carcinogenesis and progression. This same strategy improved with 2D-SDS-PAGE or HPLC, could serve as an important tool for the elucidation of novel biomarkers and/or therapeutic targets which may facilitate the clinical management of CRC patients.

## Competing interests

The authors declare that they have no competing interests.

## Authors' contributions

The work presented here was carried out in collaboration between all authors. LMR participated in the design of the study, the proteomic analysis, the results interpretation and the manuscript preparation. SVP carried out the immunohistochemical analysis and helped to draft the manuscript. EC collected the biological samples and carried out the valuation of the immunohistochemistry results. EGM and AFB carried out the design of the study, the results interpretation and the manuscript redaction. Finally, all authors have read and approved the final manuscript.

## Pre-publication history

The pre-publication history for this paper can be accessed here:

http://www.biomedcentral.com/1471-2407/11/508/prepub
